# Supplementary material for: Quality of life and well-being problems in secondary schoolgirls in Kenya: Prevalence, associated characteristics, and course predictors
Source: PLOS Glob Public Health. 2022 Dec 19;2(12):e0001338. doi: 10.1371/journal.pgph.0001338 (PMC10022324; doi:10.1371/journal.pgph.0001338)
Supplement: S1 Checklist — (DOCX) [file pgph.0001338.s001.docx]

**STROBE Statement—Checklist of items that should be included in reports of *cross-sectional studies***

|  | Item No | Recommendation |
| --- | --- | --- |
| **Title and abstract** | 1 | (*a*) Indicate the study’s design with a commonly used term in the title or the abstract |
|  |  | (*b*) Provide in the abstract an informative and balanced summary of what was done and what was found |
| Background/rationale  (Introduction paragraphs 1-5) | 2 | Explain the scientific background and rationale for the investigation being reported |
| Objectives  (Abstract paragraph 1, Introduction paragraph 6) | 3 | State specific objectives, including any prespecified hypotheses |
| Study design  (Methods paragraph 1) | 4 | Present key elements of study design early in the paper |
| Setting  (Methods paragraph 2-3) | 5 | Describe the setting, locations, and relevant dates, including periods of recruitment, exposure, follow-up, and data collection |
| Participants  (Methods paragraph 3-6) | 6 | (*a*) Give the eligibility criteria, and the sources and methods of selection of participants |
| Variables  (Methods paragraph 6-7) | 7 | Clearly define all outcomes, exposures, predictors, potential confounders, and effect modifiers. Give diagnostic criteria, if applicable |
| Data sources/ measurement  (Methods paragraph 6-8) | 8* | For each variable of interest, give sources of data and details of methods of assessment (measurement). Describe comparability of assessment methods if there is more than one group |
| Bias  (Methods paragraph 8-11) | 9 | Describe any efforts to address potential sources of bias |
| Study size  (Methods paragraph 4) | 10 | Explain how the study size was arrived at |
| Quantitative variables  (Methods paragraph 8-12) | 11 | Explain how quantitative variables were handled in the analyses. If applicable, describe which groupings were chosen and why |
| Statistical methods  (Methods paragraph 8-13) | 12 | (*a*) Describe all statistical methods, including those used to control for confounding |
|  |  | (*b*) Describe any methods used to examine subgroups and interactions |
|  |  | (*c*) Explain how missing data were addressed |
|  |  | (*d*) If applicable, describe analytical methods taking account of sampling strategy |
|  |  | (*e*) Describe any sensitivity analyses |
| Participants  (Methods Figure 1, Results paragraph 1) | 13* | (a) Report numbers of individuals at each stage of study—eg numbers potentially eligible, examined for eligibility, confirmed eligible, included in the study, completing follow-up, and analysed |
|  |  | (b) Give reasons for non-participation at each stage |
|  |  | (c) Consider use of a flow diagram |
| Descriptive data  (Results Table 1-3,  Results paragraphs 2-4) | 14* | (a) Give characteristics of study participants (eg demographic, clinical, social) and information on exposures and potential confounders |
|  |  | (b) Indicate number of participants with missing data for each variable of interest |
| Outcome data  (Results Tables 2-3)  Results paragraphs 5-6 | 15* | Report numbers of outcome events or summary measures |
| Main results  (Results Tables 4-5,  Results paragraphs 7-8) | 16 | (*a*) Give unadjusted estimates and, if applicable, confounder-adjusted estimates and their precision (eg, 95% confidence interval). Make clear which confounders were adjusted for and why they were included |
|  |  | (*b*) Report category boundaries when continuous variables were categorized |
|  |  | (*c*) If relevant, consider translating estimates of relative risk into absolute risk for a meaningful time period |
| Other analyses  (Results paragraphs 8-9, Tables S1-S7) | 17 | Report other analyses done—eg analyses of subgroups and interactions, and sensitivity analyses |
| Key results  (Discussion paragraph 1-6) | 18 | Summarise key results with reference to study objectives |
| Limitations  (Discussion paragraph 9-11) | 19 | Discuss limitations of the study, taking into account sources of potential bias or imprecision. Discuss both direction and magnitude of any potential bias |
| Interpretation  (Conclusion paragraph 1) | 20 | Give a cautious overall interpretation of results considering objectives, limitations, multiplicity of analyses, results from similar studies, and other relevant evidence |
| Generalisability  (Discussion paragraph 7-8) | 21 | Discuss the generalisability (external validity) of the study results |
| Funding  (Funder paragraph 1) | 22 | Give the source of funding and the role of the funders for the present study and, if applicable, for the original study on which the present article is based |

**Note:** An Explanation and Elaboration article discusses each checklist item and gives methodological background and published examples of transparent reporting. The STROBE checklist is best used in conjunction with this article (freely available on the Web sites of PLoS Medicine at http://www.plosmedicine.org/, Annals of Internal Medicine at http://www.annals.org/, and Epidemiology at http://www.epidem.com/). Information on the STROBE Initiative is available at www.strobe-statement.org.
