# Supplementary material for: Quality of life and well-being problems in secondary schoolgirls in Kenya: Prevalence, associated characteristics, and course predictors
Source: PLOS Glob Public Health. 2022 Dec 19;2(12):e0001338. doi: 10.1371/journal.pgph.0001338 (PMC10022324; doi:10.1371/journal.pgph.0001338)
Supplement: S1 Table — Note. a n = 2845 because school functioning school items at FU2 were no longer applicable to all respondents. (DOCX) [file pgph.0001338.s002.docx]

| Table S1. Means and standard deviations at baseline, FU1, and FU2 on the Pediatric Quality of Life inventory (PEDS-QL) | | | | | | |
| --- | --- | --- | --- | --- | --- | --- |
|  | **Baseline (n = 3998)** | | **FU1 (n = 2906)** | | **FU2 (n = 3275)** | |
| (Sub)scales | M | Sd | M | Sd | M | Sd |
| Total functioning | 75.71 | 17.61 | 84.33 | 15.17 | 86.31 ^a^ | 15.04 |
| Physical functioning | 77.50 | 21.06 | 86.45 | 17.25 | 89.37 | 16.23 |
| Emotional functioning | 73.19 | 21.55 | 81.45 | 20.84 | 83.65 | 20.62 |
| Social functioning | 79.18 | 19.17 | 88.53 | 16.99 | 90.20 | 16.49 |
| School functioning | 71.89 | 18.73 | 79.62 | 18.04 | 80.66 ^a^ | 18.60 |
| Note. ^a^ n = 2845 because school functioning school items at FU2 were no longer applicable to all respondents | | | | | | |
