# Supplementary material for: Quality of life and well-being problems in secondary schoolgirls in Kenya: Prevalence, associated characteristics, and course predictors
Source: PLOS Glob Public Health. 2022 Dec 19;2(12):e0001338. doi: 10.1371/journal.pgph.0001338 (PMC10022324; doi:10.1371/journal.pgph.0001338)
Supplement: S2 Table — Note. LL = Log-likelihood, AIC = Akaike Information Criterion; BIC = Bayesian Information Criterion LMR-LRT = Lo-Mendel-Ruben Likelihood Ratio Test. (DOCX) [file pgph.0001338.s003.docx]

| Table S2. Results of latent class analysis at baseline, FU1 and FU2 | | | | | | |
| --- | --- | --- | --- | --- | --- | --- |
|  | **LL** | **AIC** | **BIC** | **Adjusted BIC** | **Entropy** | **LMR-LRT test** |
| Baseline (n-3398) |  |  |  |  |  |  |
| one class | -70671.950 | 141359.900 | 141410.248 | 141384.828 | (-) | (-) |
| two classes | -67108.213 | 134242.425 | 134324.241 | 134282.933 | .87 | <.001 |
| three classes | -66067.747 | 132171.494 | 132284.778 | 132227.582 | .85 | <.001 |
| four classes | -65531.328 | 131108.656 | 131253.407 | 131180.323 | .86 | <.001 |
| five classes | -65322.744 | 130701.489 | 130877.708 | 130788.737 | .88 | .09 |
| FU1 (n = 2906) |  |  |  |  |  |  |
| one class | -50230.809 | 100477.618 | 100525.414 | 100499.995 | (-) | (-) |
| two classes | -47614.796 | 95255.592 | 95333.261 | 95291.955 | .92 | <.001 |
| three classes | -46935.769 | 93907.538 | 94015.080 | 93957.887 | .90 | <.001 |
| four classes | -46708.530 | 93463.060 | 93600.474 | 93527.394 | .91 | .006 |
| five classes | -46418.407 | 92892.814 | 93060.101 | 92971.135 | .94 | .191 |
| FU2 (n = 3275) |  |  |  |  |  |  |
| one class | -54509.358 | 109034.717 | 109083.469 | 109058.050 | (-) | (-) |
| two classes | -51411.022 | 102848.045 | 102927.268 | 102885.961 | .93 | <.001 |
| three classes | -50428.516 | 100893.031 | 101002.724 | 100945.530 | .93 | <.001 |
| four classes | -50063.085 | 100172.170 | 100312.334 | 100239.252 | .94 | .006 |
| five classes | -49563.600 | 99183.201 | 99353.835 | 99264.867 | .96 | .003 |
| Note. LL = Log-likelihood, AIC = Akaike Information Criterion; BIC = Bayesian Information Criterion;  LMR-LRT = Lo-Mendel-Ruben Likelihood Ratio Test | | | | | | |
