# Supplementary material for: Quality of life and well-being problems in secondary schoolgirls in Kenya: Prevalence, associated characteristics, and course predictors
Source: PLOS Glob Public Health. 2022 Dec 19;2(12):e0001338. doi: 10.1371/journal.pgph.0001338 (PMC10022324; doi:10.1371/journal.pgph.0001338)
Supplement: S3 Table — Note. LL = Log-likelihood, AIC = Akaike Information Criterion; BIC = Bayesian Information Criterion. (DOCX) [file pgph.0001338.s004.docx]

| Table S3. Results of the LTA across baseline, FU1 and FU2 (n = 3998) | | | | | |
| --- | --- | --- | --- | --- | --- |
| N classes | ***LL*** | **AIC** | **BIC** | **Adjusted BIC** | **Entropy** |
| two classes | -166026.059 | 33211.119 | 332292.632 | 332200.483 | .82 |
| three classes | -163265.576 | 326607.152 | 326846.306 | 326846.306 | .81 |
| four classes | -162233.972 | 324577.945 | 324924.090 | 324749.324 | .83 |
| Note. LL = Log-likelihood, AIC = Akaike Information Criterion; BIC = Bayesian Information Criterion | | | | | |
