# Supplementary material for: Quality of life and well-being problems in secondary schoolgirls in Kenya: Prevalence, associated characteristics, and course predictors
Source: PLOS Glob Public Health. 2022 Dec 19;2(12):e0001338. doi: 10.1371/journal.pgph.0001338 (PMC10022324; doi:10.1371/journal.pgph.0001338)
Supplement: S4 Table — (DOCX) [file pgph.0001338.s005.docx]

| Table S4. Average latent class probabilities for the most likely latent class membership at baseline, FU1 and FU2 (n = 3398) | | | | | | | | | |
| --- | --- | --- | --- | --- | --- | --- | --- | --- | --- |
|  | **Baseline** | | | **FU1** | | | **FU2** | | |
|  | Low QoL | Av. QoL | High QoL | Low QoL | Av. QoL | High QoL | Low QoL | Av. QoL | High QoL |
| Low QoL | .932 | .068 | .000 | .906 | .089 | .005 | .898 | .081 | .021 |
| Av. QoL | .059 | .884 | .057 | .084 | .824 | .092 | .040 | .897 | .063 |
| High QoL | .000 | 0.049 | .951 | .017 | .085 | .899 | .021 | .066 | .914 |
