# Supplementary material for: Quality of life and well-being problems in secondary schoolgirls in Kenya: Prevalence, associated characteristics, and course predictors
Source: PLOS Glob Public Health. 2022 Dec 19;2(12):e0001338. doi: 10.1371/journal.pgph.0001338 (PMC10022324; doi:10.1371/journal.pgph.0001338)
Supplement: S5 Table — Note. a Excluded because of multicollinearity with ’Forced sex’; BMI = Body Mass Index; SES = Socio-Economic Status; MCW versus SO = married, cohabitating, widowed versus single/other. (DOCX) [file pgph.0001338.s006.docx]

| Table S5. Odds Ratio's (95% confidence intervals) for remaining in Low QoL Group (ITT analysis: n = 1126) | | | | | | | | |
| --- | --- | --- | --- | --- | --- | --- | --- | --- |
|  | **Improved (n = 895)** | | **Not-improved (n=231)** | | **Unadjusted model** | | **Adjusted model** | |
|  | N | % | N | % | OR | 95% CI | OR | 95% CI |
| Sociodemographics |  |  |  |  |  |  |  |  |
| Age categorical (year) (n=1117) |  |  |  |  | .95 | .85-1.05 |  |  |
| <16 | 153 | 17.3 | 38 | 16.5 |  |  |  |  |
| 16 | 240 | 27.1 | 69 | 29.9 |  |  |  |  |
| 17 | 239 | 27.0 | 65 | 28.1 |  |  |  |  |
| 18 | 159 | 17.9 | 40 | 17.3 |  |  |  |  |
| 19+ | 95 | 10.7 | 19 | 8.2 |  |  |  |  |
| SES (poorest) | 421 | 47.0 | 125 | 54.1 | 1.31 | .97-1.77 |  |  |
| Marital status (MCW) | 60 | 6.7 | 16 | 6.9 | 1.05 | .58-1.89 |  |  |
| Baby at home to care for | 38 | 4.2 | 11 | 4.8 | 1.16 | .57-2.34 |  |  |
| Orphan | 26 | 2.9 | 10 | 4.3 | 1.57 | .73-3.38 |  |  |
| School and Finances |  |  |  |  |  |  |  |  |
| Missed school - all reasons | 208 | 23.2 | 60 | 26.0 | 1.18 | .83-1.66 |  |  |
| Missed school due to menstruation | 182 | 20.3 | 57 | 24.7 | 1.31 | .92-1.85 |  |  |
| Received money from boyfriend/partner | 75 | 8.4 | 20 | 8.7 | 1.04 | .59-1.86 |  |  |
| Received money from working | 159 | 17.8 | 56 | 24.2 | 1.47 | 1.03-2.08 | 1.25 | .84-1.85 |
| General Health |  |  |  |  |  |  |  |  |
| BMI categorical |  |  |  |  | 1.20 | .92-1.56 |  |  |
| Underweight (BMI<18.2) | 49 | 5.5 | 8 | 3.5 |  |  |  |  |
| Normal (BMI 18.2-25) | 686 | 76.6 | 177 | 76.6 |  |  |  |  |
| Overweight (BMI>25) | 160 | 17.9 | 46 | 19.9 |  |  |  |  |
| Drinking | 6 | 0.7 | 1 | 0.4 | .64 | .07-5.92 |  |  |
| Smoking | 1 | 0.1 | 0 | 0.0 | .00 | .00-.00 |  |  |
| Adverse Adolescent Experiences |  |  |  |  |  |  |  |  |
| Harassment for sex at school | 144 | 16.1 | 55 | 23.8 | 1.64 | 1.10-2.44 | 1.25 | .81-1.93 |
| Harassment for sex out of school | 491 | 54.9 | 141 | 61.0 | 1.30 | .99-1.69 |  |  |
| Touched indecently | 180 | 20.1 | 56 | 24.2 | 1.29 | .94-1.76 |  |  |
| Sexually active | 293 | 32.7 | 96 | 41.6 | 1.46 | 1.11-1.93 | ^a^ | ^a^ |
| Forced sex | 167 | 18.7 | 70 | 30.3 | 1.91 | 1.35-2.69 | 1.30 | .87-1.93 |
| Physical assault | 373 | 41.7 | 126 | 54.5 | 1.70 | 1.29-2.24 | 1.13 | .80-1.59 |
| Robbed | 210 | 23.5 | 74 | 32.0 | 1.56 | 1.11-2.20 | 1.09 | .73-1.62 |
| Threatened to hurt you | 313 | 35.0 | 116 | 50.2 | 1.89 | 1.45-2.46 | 1.07 | .76-1.52 |
| Threats for family to be hurt | 316 | 35.3 | 131 | 56.7 | 2.44 | 1.83-3.24 | 1.90 | 1.37-2.66 |
| Humiliation | 237 | 26.5 | 98 | 42.4 | 2.03 | 1.50-2.76 | 1.42 | .99-2.02 |
| Reproductive Health |  |  |  |  |  |  |  |  |
| HIV seropositive | 17 | 1.9 | 2 | 0.9 | 0.42 | .10-1.85 |  |  |
| HSV-2 seropositive | 150 | 16.8 | 41 | 17.7 | 1.07 | .72-1.58 |  |  |
| Early menarche <13 years) | 48 | 5.4 | 20 | 8.7 | 1.66 | .93-2.96 |  |  |
| History of pregnancy (n=338) | 37 | 12.6 | 8 | 8.5 | .71 | .34-1.47 |  |  |
| Menstruation severity |  |  |  |  | 1.02 | .77-1.34 |  |  |
| Light | 67 | 7.5 | 20 | 8.7 |  |  |  |  |
| Normal | 582 | 65.0 | 143 | 61.9 |  |  |  |  |
| Heavy | 246 | 27.5 | 68 | 29.4 |  |  |  |  |
| Menstruation duration (n=1102) |  |  |  |  | 1.07 | .77-1.49 |  |  |
| <3 days | 32 | 3.7 | 11 | 4.9 |  |  |  |  |
| 3-5 days | 679 | 77.5 | 166 | 73.5 |  |  |  |  |
| >5 days | 165 | 18.8 | 49 | 21.7 |  |  |  |  |
| Menstruation stopped activities | 349 | 39.0 | 111 | 48.1 | 1.48 | 1.10-1.98 | 1.16 | .83-1.63 |
| No sanitary pads | 416 | 46.5 | 109 | 47.2 | 1.03 | .76-1.39 |  |  |
| Had to do something to get sanitary pads | 179 | 20.0 | 63 | 27.3 | 1.49 | 1.08-2.04 | 1.07 | .75-1.52 |
| Note. ^a^ Excluded because of multicollinearity with 'Forced sex'; BMI = Body Mass Index; SES = Socio-Economic Status;  MCW versus SO = married, cohabitating, widowed versus single/other | | | | | | | | |
