# Supplementary material for: Quality of life and well-being problems in secondary schoolgirls in Kenya: Prevalence, associated characteristics, and course predictors
Source: PLOS Glob Public Health. 2022 Dec 19;2(12):e0001338. doi: 10.1371/journal.pgph.0001338 (PMC10022324; doi:10.1371/journal.pgph.0001338)
Supplement: S6 Table — Note; a Excluded because of multicollinearity with ’Forced sex’; BMI = Body Mass Index; SES = Socio-Economic Status; MCW versus SO = married, cohabitating, widowed versus single/other. (DOCX) [file pgph.0001338.s007.docx]

| Table S6. Odds Ratio's (95% confidence intervals) for remaining in Low QoL Group (completers analysis: n = 794) | | | | | | |
| --- | --- | --- | --- | --- | --- | --- |
|  | **Improved (n=603)** | | **Not-improved (n=191)** | | **Unadjusted model** | **Adjusted model** |
|  | N | % | N | % | OR (95%CI) | aOR (95% CI) |
| Sociodemographics |  |  |  |  |  |  |
| Age categorical (year) (n=792) |  |  |  |  | .99 (.87-1.13) |  |
| <16 | 104 | 17.3 | 29 | 15.2 |  |  |
| 16 | 160 | 26.6 | 57 | 29.8 |  |  |
| 17 | 176 | 29.3 | 53 | 27.7 |  |  |
| 18 | 100 | 16.6 | 35 | 18.3 |  |  |
| 19+ | 61 | 10.1 | 17 | 8.9 |  |  |
| SES (poorest) | 296 | 49.1 | 104 | 54.5 | 1.21 (.87-1.69) |  |
| Marital status (MCW) | 36 | 6.0 | 14 | 7.3 | 1.29 (.67-2.49) |  |
| Baby at home to care for | 26 | 4.3 | 10 | 5.2 | 1.25 (.58-2.71) |  |
| Orphan | 12 | 2.0 | 10 | 5.2 | 2.74 (1.13-6.65) | 3.17 (1.25-8.02) |
| School and finances |  |  |  |  |  |  |
| Missed school - all reasons | 139 | 23.1 | 51 | 26.7 | 1.23 (.84-1.81) |  |
| Missed school due to menstruation | 124 | 20.6 | 49 | 25.7 | 1.36 (.92-2.00) |  |
| Received money from boyfriend/partner | 44 | 7.3 | 18 | 9.4 | 1.32 (.68-2.55) |  |
| Received money from working | 107 | 17.7 | 48 | 25.1 | 1.56 (1.06-2.29) | 1.24 (.80-1.93) |
| General Health |  |  |  |  |  |  |
| BMI categorical |  |  |  |  | 1.18 (.85-1.64) |  |
| Underweight (BMI<18.2) | 27 | 4.5 | 7 | 3.7 |  |  |
| Normal (BMI 18.2-25) | 469 | 77.8 | 145 | 75.9 |  |  |
| Overweight (BMI>25) | 107 | 17.7 | 39 | 20.4 |  |  |
| Drinking | 2 | 0.3 | 0 | 0.0 | .00 (.00 -.00) |  |
| Smoking | 0 | 0.0 | 0 | 0.0 | na |  |
| Adverse Adolescent Experiences |  |  |  |  |  |  |
| Harassment for sex at school | 95 | 15.8 | 45 | 23.6 | 1.64 (1.05-2.58) | 1.22 (.74-1.99) |
| Harassment for sex out of school | 333 | 55.2 | 119 | 62.3 | 1.33 (.96-1.84) |  |
| Touched indecently | 114 | 18.9 | 45 | 23.6 | 1.33 (.94-1.88) |  |
| Sexually active | 186 | 30.8 | 83 | 43.5 | 1.70 (1.24-2.33) | ^a^ |
| Forced sex | 111 | 18.4 | 64 | 33.5 | 2.22 (1.53-3.20) | 1.65 (1.08-2.52) |
| Physical assault | 240 | 39.8 | 103 | 53.9 | 1.77 (1.28-2.44) | 1.12 (.74-1.70) |
| Robbed | 135 | 22.4 | 62 | 32.5 | 1.69 (1.16-2.47) | 1.33 (.86-2.06) |
| Threatened to hurt you | 206 | 34.2 | 94 | 49.2 | 1.86 (1.38-2.50) | .96 (.62-1.46) |
| Threats for family to be hurt | 213 | 35.3 | 109 | 57.1 | 2.45 (1.78-3.36) | 1.92 (1.34-2.75) |
| Humiliation | 166 | 27.5 | 85 | 44.0 | 2.07 (1.48-2.89) | 1.40 (.94-2.08) |
| Reproductive Health |  |  |  |  |  |  |
| HIV seropositive | 8 | 1.3 | 1 | 0.5 | .40 (.05-3.37) |  |
| HSV-2 seropositive | 105 | 17.4 | 34 | 17.8 | 1.01 (.65-1.57) |  |
| Early menarche <13 years) | 35 | 5.8 | 15 | 7.9 | 1.37 (.71-2.64) |  |
| History of pregnancy (n=269) | 26 | 13.9 | 7 | 8.5 | .61 (.26-1.46) |  |
| Menstruation severity |  |  |  |  | 1.06 (.79-1.44) |  |
| Light | 42 | 7.0 | 17 | 8.9 |  |  |
| Normal | 398 | 66.0 | 114 | 59.7 |  |  |
| Heavy | 163 | 27.0 | 60 | 31.4 |  |  |
| Menstruation duration (n=779) |  |  |  |  | .94 (.67-1.33) |  |
| <3 days | 20 | 3.4 | 11 | 5.9 |  |  |
| 3-5 days | 446 | 75.5 | 135 | 71.8 |  |  |
| >5 days | 125 | 21.2 | 42 | 22.3 |  |  |
| Menstruation stopped activities | 237 | 39.3 | 97 | 50.8 | 1.65 (1.17-2.34) | 1.29 (.87-1.90) |
| No sanitary pads | 285 | 47.3 | 94 | 49.2 | 1.09 (.79-1.50) |  |
| Had to do something to get sanitary pads | 123 | 20.4 | 54 | 28.3 | 1.53 (1.07-2.19) | 1.07 (.72-1.60) |
| Note; ^a^ Excluded because of multicollinearity with 'Forced sex'; BMI = Body Mass Index; SES = Socio-Economic Status;  MCW versus SO = married, cohabitating, widowed versus single/other | | | | | | |
