# Supplementary material for: Quality of life and well-being problems in secondary schoolgirls in Kenya: Prevalence, associated characteristics, and course predictors
Source: PLOS Glob Public Health. 2022 Dec 19;2(12):e0001338. doi: 10.1371/journal.pgph.0001338 (PMC10022324; doi:10.1371/journal.pgph.0001338)
Supplement: Attachment — Submitted filename: 140822_Rebuttal_QoL 15Aug22.docx [file pgph.0001338.s008.docx]

**Reviewer #1:**

The article is written in a plain English language. The general objective/aim is presented at the end of the introduction. The “material and method” presents the research procedure, but with an unnecessarily detail.

*We shortened this section (see point # 3 concerning Results of Rev. # 1 below)*

Results are presented in the tables and figures, which demonstrates n, %, OR and 95% CI. Additional information such as funding and competing interest are provided. The scales used are widely used elsewhere. Next I justify the answers to the review questions. There are some grammatical and spelling errors that needs to be addressed thoroughly in revised manuscript.

*Thank you, the manuscript has been carefully reviewed by co-authors whose mother tongue is English.*

I. Regarding question 1, I replied “partly” because the tested hypotheses are not presented early in the introduction, nor it is presented elsewhere in the manuscript. Tested hypothesis should be presented early in the manuscript, so that the reader can analyze and compare results presented, accordingly.

*We now provide the tested hypotheses (see point #7 concerning Introduction of Rev. # 1 below)*

II. Regarding question 3, the original dataset is not uploaded, however, the data points, behind means, medians, IQR, are available.

Some additional points to be considered while revising the manuscript is detailed below.

*Introduction*
1. It was better to write the median point prevalence rate (IQR) were … . the IQR is missing in the parenthesis for anxiety.

*This has been changed as recommended.*

2. “Included studies in this and a previous systematic review”, it is not clear what does the word “this” refer to. Although one can imagine that “this” may refer to the reference number 4, but is not scientifically correct to start a paragraph with a demonstrative pronoun that came in the previous paragraph.

*This has been changed as recommended.*

3. I believe references 6, 7, 8, 9, 10 and 11 could be combined and shortened. They reveal similar findings (girls showing higher rates of depressive syndromes than boys). Writing above references in detail made the introduction section too long.

*We agree and greatly reduced this paragraph by no longer describing the individual studies.*

*The paragraph now reads:*

*"In Kenya, few studies have focused on mental health in adolescents in normative environments such as secondary schools [6-11]. Prevalence rates of anxiety and depression symptoms and syndromes are elevated but vary between studies depending on the instruments and cut-off points used. Most studies report more emotional and behavioral problems in girls compared to boys [6, 8-11]. Furthermore, relationships with older age [8, 1], overcontrolling parental behaviour [7, 9], being on a boarding school [9], and belonging to a minority tribe are reported [10]."*

4. The paragraph that started at the bottom of page 5 and continued to middle of page 6, is too clumsy, has grammatical errors, and a vague presentation of data. The author pointed to the QoL and presented a definition, but failed to provide a reference. At the end of this paragraph, authors compared the findings of reference 12 with another study, which is not a scientific practice, in the introduction.

*We adapted this paragraph as below. By also introducing sexual and reproductive health harms in relation to girls' QoL we could also formulate our study hypotheses at the end of the introduction more clearly.*

*"Mental health is more than the absence of mental disorders. Individuals' view of their own quality of life (QoL) offers a broader conceptualization of health and may yield a useful additional indicator of mental health [12]. QoL encompasses a subjective evaluation of an individual’s well-being as well as objective descriptions of individuals and their circumstances that are associated with subjective well-being. Restricting ourselves to subjective child and adolescent QoL, it comprises subjective dimensions such as the experience of physical, psychological, social, and school functioning [12]. As far as we know, only two studies have assessed QoL in children and adolescents in sub-Saharan Africa [12,13]. Both studies reported relatively high levels of QoL and noted significant associations between QoL and mental health problems. Girls reported lower levels of QoL on all dimensions compared with boys [12]. The lower QoL in girls may be due to exposure to sexual and reproductive health harms, which are disproportionately high among adolescent girls in sub-Saharan Africa [14].*

5. The last paragraph of introduction starts with the phrase “in conclusion”. This is not a good place for this phrase, as the introduction section does not have a concluding statement.

*This has been adapted accordingly*

6. The aim of the study could have been presented as “the aim of this study is to assess prevalence, associated characteristics and predicting factors of QoL and well-being problems among schoolgirls in Kenya.”

*This has been adapted accordingly*

7. The hypothesis of the study is not presented in the introduction. It is a scientific practice to present hypothesis in the introduction, so that readers understand what they are looking for.

*We now present the following hypotheses at the end of the introduction:*

*" The aim of this study was to assess the prevalence, associated characteristics, and predictive factors of QoL among schoolgirls in Kenya. It was hypothesized that a substantial subgroup of schoolgirls would report a low QoL, that schoolgirls with a low QoL would report more adverse adolescent experiences and sexual and reproductive health issues, and that these negative experiences and health issues would be predictive of a persistent low QoL."*

Materials and methods
8. In “study area and population”, in line 2, “in” is written twice.

*Edited as suggested.*

9. In “Study recruitment and procedures” the word “ministry” is not necessary at the beginning of line 4.

*This has been removed.*

10. In “Study recruitment and procedures” it is better to refer the reader to original reference and not include all details in the current manuscript. (Study samples were recruited as mentioned elsewhere [14]”.

*Edited as suggested.*
11. In “Study recruitment and procedures” references 16, 17, and 18 are missing. Authors failed to provide references in a consecutive order. Reference 15 has been mentioned previously, in “Study area and population”; then authors suddenly jumped to reference 19, without mentioning above mentioned missing references.

*This inconsistency has been solved by referring the reader to the original reference for study recruitment and procedures and no longer applies.*

12. In “Outcome measurements”, when reference 16 and 17 are provided, reader can refer to them and it is not necessary to provide details of the instruments again in the current manuscript. In fact, the essence of referencing is to avoid repetition of information between literature.

*We agree with the reviewer that it is not necessary to provide details on instruments that can be found in the literature elsewhere (including the CCP trial paper). To shorten this paragraph we deleted general information about the PedsQL (i.e., that Likert scaled item answers were reverse scored and linearly transformed with higher mean scores indicating better well-being). However, given the discussion on the transculturally applicability of 'western' standardized self-report measures we would like to retain the short description of our outcome measures as the psychometric characteristics (Cronbach's alpha) of the PedsQL and PHQ-9 in the present study were good to excellent and have not been reported previously. We come back to this important topic in the study limitations section of the discussion.*

13. In “Outcome measurements”, it is better to provide zero before a decimal number (for example range 0.68 - 0.89).

*Edited accordingly*

14. In “risk factors”, line 3 “SES” should be written in full, in the first mention.

*Edited accordingly*

15. Line 4, page 11, “observes” should be changed to “observed”.

*Edited accordingly*

*Results*

1. Figure 2 is labelled as figure 1, at the end of the manuscript.

*As Figure 1 has now been removed Figure 2 is now Figure 1 in the text of the manuscript.*

2. Description of table 1 does not clearly demonstrate what actually is in the Table. The High QoL, Average QoL, and Low QoL were not clearly defined in Table 1. Cross-ref of the information and Table 1, is very difficult.

*This table has been removed and the necessary information of this table is incorporated in the text of the manuscript.*

3. Many overlapping information and data are presented in the Results section, that actually belong to the Material and Methods section. These really need to be controlled and adjusted in the revised version.

*We agree that the structure of the Results section could be greatly improved and that redundant or unnecessary information in Materials and Methods and Results sections could be removed. As suggested by the reviewer the main part of the Results section is now devoted to three topics (Prevalence of QoL and well-being problems, Associated characteristics of QoL problems, and Predicting factors of QoL problems) corresponding to the three main research questions. The section begins with a subsection on Participants (one paragraph) and Preliminary analyses (two paragraphs).*

*I*n this latter section we succinctly describe the most pertinent results of the Latent Transition Analysis and subsequent formation of the three QoL groups. In our opinion it is necessary to preserve the description of these analyses as they form a steppingstone for answering the three main research questions and transparency and reproducibility of this study would be compromised by not detailing the LTA results in the main body of the text and supplementary material.

*These changes have resulted in a more concise and better structured text, as well as the removal of two tables (original Table 1: Latent status prevalence and transition matrix estimates over three time points according to Latent Transition Analysis and combining original Tables 2 and 3) and one figure (original Figure 1: the flow diagram of the CCG trial participant recruitment).*

4. The headings of table 3 could be modified as OR (95% CI), beneath which both columns could be joined to shrink the size of the table. For example, for baseline data, mobility problem it could be written 7.13 (5.92-8.58). this way data could be more readable, and clearly presented.

*Edited accordingly*

5. Table 4, it is better to combine N and % in one column as “N (%)”. This way, the table looks neater and more readable.

*Edited accordingly*

6. Table 4 is confusing, for example under the age categories (years), <16, 16 … looks to be in a separate column, with no heading. It is better to re-organize the table to be more readable. Moreover, presentation of data is not straightforward. For example, for “SES (poorest vs less poor)” one cannot understand the corresponding numbers and percentages are for those who are poorest, or those who are less poor; and for “Marital status (MCW/SO)” similar problem exists. It is also better to write the number of participants corresponding to each variable next to it, not in the footnote of the table.

*Adapted accordingly*

7. Table 5 and Table 6, similar to Table 3, it is better to combine OR and 95% CI in the same column as OR (95% CI). This way, data will be presented neatly and more readably. I also believe that if authors used Maximum Likelihood ratio model, it was much better to show only those variables that were significantly predicting high QoL. Furthermore, the significance value (p value) is not provided, one cannot understand which factors are considered associated or predicting factors for QoL.

*Edited accordingly*

8. The result section, overall, is presented in a way that is not easy to understand, contains not so much relevant information, and overlapping data/information from the Material and Method section. If authors focused only on three components of their study question/aims, the Results was more structured and reader-friendly. I believe authors only needed to report 1) prevalence 2) associated factors, and 3) predicting factors of low QoL and well-being among participants. Other Tables, Figures, and Supplementary material were not required. Authors, I believe, mixed the two projects (CCG and current study question) together, which caused writing a long manuscript with information and data jumping from one matter to the next. I recommend that authors more focus on the study question and re-write the Results section only on prevalence, associated factors and predicting factors of low QoL and well-being. Much of the steps involved included in the Results section, are compulsory initial steps of a research, not required to report.

*Edited accordingly (see also point # 3 f Rev. # 1 above)*

9. Figures are not readable and should be drawn afresh.

*Figure 1 has been redrawn as requested*

*Discussion*

1. Reference 26 refers to a study that was conducted in California, USA. But authors incorrectly referred to it as most high- and middle-income countries. One expects when the word “most” appears, authors should provide many references or at least a systematic review/meta-analysis study.

*We agree and now specify that QoL scores were in the range of those 'from large standardization samples in the United States.'*

2. Authors wrote “QoL is negatively related to both the presence and the severity of psychopathology [27]”. But reference 27 refers to a study that only focused on “mood-related disorders”. Although mood-disorders are a type of psychopathology, it is not the only one. Therefore, it is better to clearly write ““QoL has been negatively related to both the presence and the severity of mood-related disorders [27]”.

*We agree and changed the text accordingly.*

3. In the last part of the first paragraph in Discussion, author concluded their findings, but also provided references. If it is what authors believe that came from their study, no reference is required. If it is similar to what others found and concluded, then this paper is not really “original”.

*We thank the reviewer for these observations, which make us realize that we insufficiently described the originality of the data on prevalence of QoL problems by using a person-centered (instead of a variable-centered) approach. To make this clearer we rewrote the first two paragraphs of the discussion as follows:*

"Our first research question was to determine the extent of QoL problems among adolescent secondary schoolgirls in rural Kenya. In the total sample, the mean level of QoL proved to be well within the range of those from large standardization samples in the United States [26]. These results concur with those of two previous variable-centered studies among sub-Saharan adolescents [12,13], also showing that overall the adolescent-reported mean level of QoL is relatively high. However, using a person-centered approach, we were able to show that mean scores for the whole sample may be misleading as we could identify a latent class with 28% of girls reporting substantially lower levels of QoL.

Previous studies have found that QoL is negatively related to both the presence and the severity of mood-related disorders [27], and PEDS QoL scores have been found to reflect changes in depression severity [28]. Also, studies among sub-Saharan adolescents reported that QoL is affected by mental health issues [12,13]. Similarly, girls from our lower QoL group reported psychological health issues in particular (such as a higher prevalence of probable severe depression, not feeling happy at home or school, feelings of pain/distress, and anxiety/depression). As QoL and mental health problems are intrinsically intertwined, mental health practitioners in Kenya can use QoL scores to gauge the potential effects of problems on the mental well-being of children and youth referred to their practice [12]. The 28 percent of schoolgirls in the Low QoL group falls well within the range of prevalence rates of child and adolescent common mental health problems in sub-Saharan Africa as reported in previous studies among normative samples [4]."

4. An incorrect use of writing signposts (for example, “Finally”) appears frequently in the discussion as well as introduction sections.

*These have been removed*

5. Grammatical error “As far as we are aware, is this one of the first studies looking longitudinally at predictors of persistence of QoL and well-being problems in sub-Saharan adolescents”.

*Corrected*

6. The first paragraph on page 37 looks irrelevant to study subject.

*We agree and removed this first paragraph.*

7. Paragraph 2 and 3 on page 37 should go under “Limitation” heading.

*Adapted accordingly*

8. In the last sentence of Discussion on page 38, authors indicated “Lastly, unobserved confounding precludes drawing strong causal inferences from observational data.” This means that even authors themselves do not rely on the results of their study to reveal any causal relationship between associated characteristics and low QoL and well-being.

*As observational research in general is in principle prone to unobserved confounding precluding strong causal inferences and we do not have ideas about specific confounding variables in the present study, we deleted this last sentence.*

**Reviewer #2:**

Congratulations to the authors for the article on this very important topic and about which there is still much to understand.
Overall, the paper is well done, giving the fact that there is limited data in this area of research in Sub-Saharan Africa.

*Gratefully acknowledged*

That notwithstanding, I have some few comments that needs to be addressed by authors.

First, the 3rd sentence under introduction section, “Adolescents living in low-and middle income countries (LMIC) are disproportionately affected, and the proportion of disease burden in children and youth attributable to mental and substance use disorders will increase as reproductive health and the management of infectious diseases improve in LMICs [3].” This statement doesn’t seem clear to me and authors need to review to maintain the intended meaning.

*We rephrased this sentence as follows to make the meaning more clear:*

*"The* *literature to date suggests that the proportion of disease burden in children and youth attributable to mental and substance use disorders is smaller in low-and middle-income countries (LMIC) due to higher rates of other ongoing diseases. This burden will increase as reproductive health and the management of infectious diseases improve in LMICs [3]."*

Secondary, could the authors explain why they opted to include only girls’ day schools and any special reason(s) for excluding boarding schools.

*Boarding schoolgirls were excluded from the trial because their exposure to challenges of daily life, including risks related to sexual and reproductive health, are minimal in boarding schools, where girls stay within the school boundary for the whole term. We have edited the limitations section on this.*

**Reviewer #3:**

The essence of the manuscript is an important public health concern. However, there is need to rewrite the paper for a better reading and understanding. Statement like "have serious QOl and well-being is confusing and this appeared in several areas.
Also, the introduction, results, discussion and conclusion will need extensive revision

*As Rev. # 3 gives no specific comments or suggestions we found it hard to include these review comments in our revision.*

*We hope that by incorporating the more specific comments and suggestions of Rev. # 1 and #2 we have been able also to address his or her comments regards our paper.*

*We agree that that the term 'QoL and well-being' may be confusing, we now consistently use the term 'QoL’.*

*The strong relationships between quality of life, EQ-5D-3L, PHQ-9 scores, and self-rated happiness is now only referred to in the validation section of QoL groups and in the discussion. We hope that the text is now clearer.*
